# Effect of acid washing and torrefaction combined pretreatment on the properties of waste tobacco stem biomass and the quality of pyrolysis bio-oil

**DOI:** 10.3389/fchem.2025.1603584

**Published:** 2025-06-11

**Authors:** Anni Wang, Jianhang Hu, Long Zhang, Hua Wang

**Affiliations:** ^1^ Faculty of Metallurgical and Energy Engineering, Kunming University of Science and Technology, Kunming, China; ^2^ Engineering Research Center of Metallurgical Energy Conservation and Emission Reduction, Ministry of Education, Kunming University of Science and Technology, Kunming, China; ^3^ State Key Laboratory of Complex Nonferrous Metal Resources Clean Utilization, Kunming University of Science and Technology, Kunming, China; ^4^ Hefei Institute of Physical Science, Chinese Academy of Sciences, Hefei, China

**Keywords:** waste tobacco stem, torrefaction, acid washing, combined pretreatment, pyrolysis, bio-oil

## Abstract

Aiming at the issues of low yield and poor quality of bio-oil obtained from direct pyrolysis of biomass, in this study, waste tobacco stems (TS) were used as raw materials, and the pretreatment methods of torrefaction and acid washing were adopted to study the effects of different torrefaction temperatures and pretreatment sequences on the quality of TS biomass and bio-oil. Results showed that combined pretreatment synergistically integrated deoxygenation from torrefaction and deashing from acid washing. High-temperature torrefaction-based combined pretreatment reduced TS oxygen content from 54.83% to 20.14%. Acid washing pretreatment achieved more than 90% removal of inorganic elements (K, Cl and Mg). The order of combined pretreatment also had an important influence on biomass pyrolysis. Torrefaction-acid washing pretreatment decreased ash content of TS, increased the relative content of sugars and aromatic compounds in bio-oil, reduced alcohols and ketones relative contents, and improved bio-oil higher heating value (HHV) and pH. Acid washing-torrefaction pretreatment enhanced bio-oil productivity, increased nitrogen-containing compounds and phenols relative content, reduced acids and aldehydes contents, and lowered bio-oil water and ash contents. Additionally, with the increase in torrefaction temperature, the O/C molar ratio of TS decreased, the HHV of TS increased, and the thermal cracking of nicotine in bio-oil to generate pyridine compounds was promoted. This study demonstrates a viable pathway to convert TS into high-quality fuels and bio-oil via combined pretreatment, offering new insights for optimizing biomass pyrolysis technology and enhancing resource utilization efficiency.

## 1 Introduction

Faced with the dual challenges of global energy transformation and ecological protection, biomass resources have become an important source of raw materials for the production of clean energy and high-value chemicals because of their renewable and environmentally friendly characteristics (Hu and Gholizadeh, 2020). As one of the main non-food crops, tobacco plays an important role in agriculture (Banožić, et al., 2020). China is one of the world’s largest tobacco producers, with an annual output of 4-5 million tons of tobacco, and a large number of tobacco processing by-products are incinerated and buried every year, resulting in serious waste of resources and environmental pollution (Ma et al., 2023a). It is reported that the global tobacco growing and cigarette manufacturing industry generates up to 200 million tons of tobacco waste every year (Tian et al., 2023). Among them, tobacco stems (TS), as a part of tobacco waste, account for about 25%–30% of the quality of tobacco leaves, and its compounds (nicotine, solanitol, protein) have unique value, in-depth study of these compounds, on the one hand, can improve the quality fraction of nicotine and flavor in tobacco products, on the other hand, it also provides a preferred raw material for the preparation of recycled tobacco leaves (Cai et al., 2016). Therefore, the development of efficient and environmentally friendly tobacco waste recycling technology is of great significance for energy utilization optimization and environmental sustainable development.

Biomass pyrolysis refers to the process of converting biomass into biochar, bio-oil and pyrolysis gas at high temperatures (300°C–900°C) and anaerobic conditions (Yang et al., 2021). Among them, pyrolysis liquefaction is an effective way to promote the recycling of tobacco waste, and the liquid products obtained can be used as chemical raw materials and biomass fuels. However, the application potential of liquid products obtained by direct pyrolysis is significantly affected by the properties of raw materials, and there are problems such as high oxygen content, acid content and ash content, resulting in poor thermal stability, low calorific value, strong corrosion and high viscosity, which seriously limits its practical application (Peng et al., 2022; Zhang et al., 2018a). At present, the research on tobacco waste resource pyrolysis mainly focuses on the effects of raw material type, pyrolysis kinetics and pyrolysis conditions (temperature, heating rate, gas atmosphere) on the composition of pyrolysis products and reaction mechanism. However, the research on pretreatment methods before pyrolysis to improve the quality of bio-oil is limited (Bai et al., 2023; Chen et al., 2024; Ma et al., 2023b). Therefore, it is of great practical significance to develop efficient pretreatment technology to improve the yield and quality of bio-oil.

Studies have shown that torrefaction pretreatment at a lower temperature (200°C–300°C), under normal pressure and inert conditions can improve the energy density, calorific value and grinding performance of biomass, while effectively reducing the water content and acidic composition of bio-oil (Fu et al., 2022; Abelha et al., 2019). (Zhang et al., 2018b) reported that after torrefaction rice husks at 270°C, the oxygen content of biomass decreased from 40.44% to 21.59%. This was due to thermochemical reactions such as dehydration, dehydroxylation and decarboxylation that occurred during the roasting process, and meanwhile, torrefaction also increased the HHV of biomass and bio-oil. However, torrefaction pretreatment alone has some limitations. For example, Gao et al. (2022) discovered that torrefaction could not solve the problems caused by alkali and alkaline earth metal elements (AAEMs), which promote the secondary cracking of volatiles and thus reduce the bio-oil yield. Hu et al. (2023) found that the presence of AAEMs would increase the content of oxygen-containing compounds such as ketones and acids in bio-oils, and significantly increase the yield of biochar. Therefore, it is important to remove AAEMs from biomass feedstock before thermochemical conversion to improve the quality of bio-oil.

In recent years, acid washing, as an effective pretreatment method, has shown good results in removing AAEMs from biomass and improving bio-oil yield (Liu et al., 2022a; Ma et al., 2023c). However, the use of inorganic acids (hydrochloric acid, sulfuric acid, phosphoric acid, etc.) to treat biomass may incorporate unnecessary elements (Cl, S, P, etc.) into the pyrolysis products and cause environmental pollution (Cen et al., 2019). In addition, the use of strong acid treatment may destroy the cell wall structure of biomass, thus affecting the subsequent pyrolysis characteristics (Jiang et al., 2013). In contrast, mild organic acids (such as formic acid, acetic acid) have the advantages of low corrosion to equipment, easy handling, and less pollutant emissions. Cen et al. (2019) found that acetic acid in the aqueous-phase bio-oil could effectively remove AAEMs from biomass, significantly increasing the yield and HHV of bio-oil. Cao et al. (2019) reported that washing algal biomass with dilute acid could increase the yields of bio-oil and non-condensable gas, decrease the yield of biochar, and that acid washing broke the hydroxyl bonds, reducing the contents of phenols and carboxylic acids in bio-oil and increasing the content of aliphatic hydrocarbons. Compared with water washing pretreatment, pyrolysis after acid washing can obtain higher yields of bio-oil and sugars from biomass (Cen et al., 2019; Liu et al., 2022b; Oudenhoven et al., 2016). The pretreatment of peanut shells by washing with hydrochloric acid and acetic acid, followed by subsequent catalytic pyrolysis, can significantly increase the yield of aromatics in bio-oil (Li et al., 2022). (Cen et al. 2025) further confirmed that acid washing could reduce the ash content in biochar and significantly improve the removal rate of metal species.

At present, torrefaction and acid washing have been widely used in biomass pretreatment, but there are few studies on the effects of the two combined applications and different pretreatment sequences on biomass and its pyrolysis products. Based on this, the effects of the combined pretreatment methods of “torrefaction - acid washing” and “acid washing - torrefaction” on the yield of pyrolysis products at different torrefactioning temperatures (220, 260°C and 300°C) were systematically investigated by using a fixed bed pyrolysis reactor. Meanwhile, the regulation mechanism of different pretreatment conditions on physicochemical properties of TS and its bio-oil was studied by means of ICP-OES and GC/MS. The aim of this study is to improve the resource utilization efficiency of tobacco waste, transform TS waste into upgraded fuels and high-value chemicals, and provide scientific basis for the development and optimization of biomass pyrolysis technology.

## 2 Materials and methods

### 2.1 Materials and instruments

TS was sourced from a Yunnan tobacco processing factory. Before the experiment, TS was dried in a drying oven at 105°C for 24 h, and after crushing, the TS particles were sieved to 0.425 mm–0.250 mm (40–60 mesh) for use. The acetic acid used was provided by Tianjin Shentai Chemical Reagent Co., Ltd. Analytical reagent.

Horizontal Tube Furnace (GSL-1100X-S, China); Vertical Tube Furnace (OTF-1200X, China); Constant Temperature Magnetic Stirrer (HJ-6B, China); Electric Thermostatic Air-Blast Drying Oven (DHG-9023A, China); Gas Chromatograph Mass Spectrometer (GC-MS) (8860/5977A, United States); Elemental Analyzer (Vario EL III, Germany); Inductively Coupled Plasma-Optical Emission Spectrometer (ICP-OES) (iCAP 7,400, United States), pH Meter (pHS-3C, China); Calorimeter (SDACM3000, China); Electronic Balance (Sensitivity 0.000 1 g, AUW320, Japan).

### 2.2 Pretreatment procedures

Torrefaction pretreatment: The torrefaction was conducted in the horizontal tube furnace of which setting temperatures were 220°C, 260°C, and 300°C. The carrier gas was high-purity nitrogen (99.999%) with a flow rate of 100 mL/min. The tube furnace was heated to the target temperature at a rate of 10 °C/min. Subsequently, a quartz boat containing 10 g of TS samples was placed at the center of the reactor for torrefaction, which was maintained continuously for 1 h. After torrefaction, the samples were removed from the heating furnace and naturally cooled to room temperature. Finally, the TS pretreatment samples under different torrefaction conditions were obtained.

Acid-Washing pretreatment: A 4 wt% acetic acid solution was first prepared for the acid-washing process. 10 g of TS samples were immersed in 200 mL of this solution and stirred continuously at 30°C using a magnetic stirrer for 3 h. After stirring, the solution was allowed to settle for 30 min, after which the filter cake was thoroughly washed with deionized water until the filtrate reached near-neutral pH. The washed filter cake was then dried in an oven at 105°C for 12 h, yielding acid-pretreated TS samples.

**FIGURE 1:**
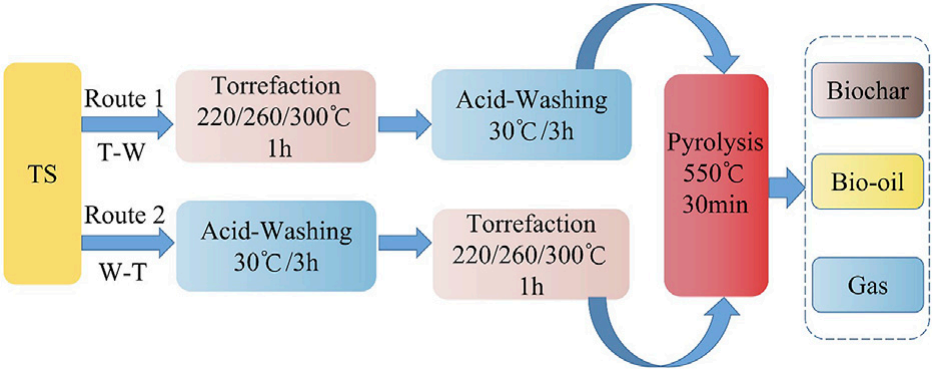
Combined pretreatment and pyrolysis process of TS biomass.

Combined pretreatment: Figure 1 illustrates two pretreatment pathways, “Torrefaction-Acid Washing-Pyrolysis” and “Acid Washing-Torrefaction-Pyrolysis”. The pretreatments involved combining acid washing with torrefaction at varying torrefaction temperatures (220°C, 260°C, and 300°C), and adjusting the sequence of these steps to prepare TS. Subsequently, the pretreated samples were subjected to pyrolysis at 550°C, resulting in the production of bio-oil, biochar, and pyrolysis gas.

### 2.3 Sample label

Table 1 enlists the labels of the samples and the pretreatment methods. Untreated tobacco stem raw material is designated as TS. The labels L, M, and H denote light, medium, and high torrefaction intensities, respectively. Tobacco stem samples pretreated with torrefaction at various temperatures are accordingly labeled LTS, MTS, and HTS. Samples treated with acetic acid washing are labeled WTS. For combined pretreatments, samples undergoing acid washing followed by torrefaction are labeled W-LTS, W-MTS, and W-HTS, whereas those treated in the reverse sequence, with torrefaction followed by acid washing, are labeled as LTS-W, MTS-W, and HTS-W.

**TABLE 1:** The labels of samples and their pretreatment methods.

Sample and pretreatment			Label
Sample	Tobacco stem	Raw	TS
Single pretreatment	Torrefaction	220°C	LTS
		260°C	MTS
		300°C	HTS
	Acid washing	Acetic acid (4 wt%)	WTS
Combined pretreatment	Torrefaction - Acid washing	220°C	LTS-W
		260°C	MTS-W
		300°C	HTS-W
	Acid washing - Torrefaction	220°C	W-LTS
		260°C	W-MTS
		300°C	W-HTS

### 2.4 Pyrolysis experiments

**FIGURE 2:**
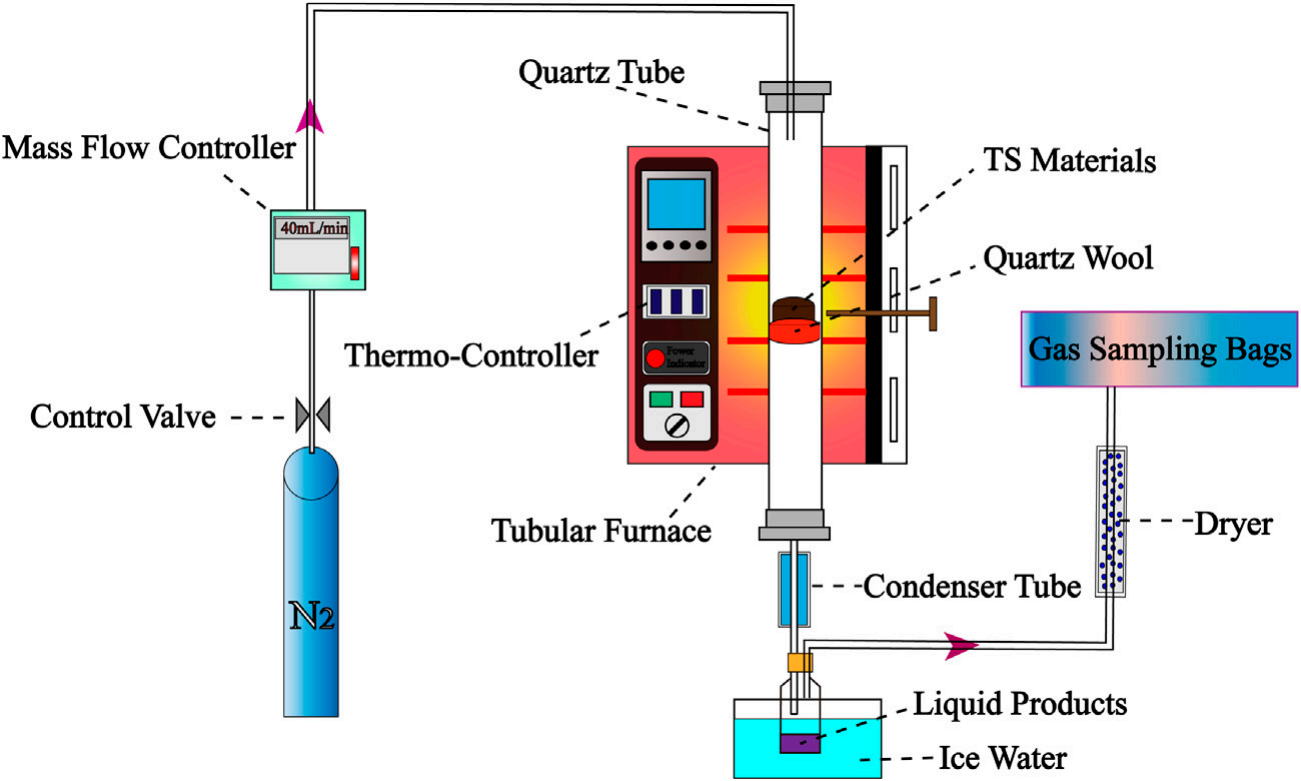
Experimental apparatus for pyrolysis.

The pyrolysis experimental device, as shown in Figure 2, mainly includes a temperature control system, a flow control system, a reaction system and a product collection system. A quartz tube with an inner diameter of 13.5 mm and a length of 1,050 mm was used as the pyrolysis reactor in the reaction system. The product collection system consisted of a liquid product collector, a dryer and a gas collection bag.

Before the experiment, high purity nitrogen (99.999%) was injected into the reactor at a flow rate of 40 mL/min to ensure that the system maintained an inert atmosphere. For each experiment, 5 g pre-treated samples were weighed and placed in the center of a quartz tube supported by quartz cotton. The vertical tube furnace was heated to 550°C at a heating rate of 15°C/min, and the quartz tube loaded with samples was quickly inserted into the reactor and kept for 30 min for pyrolysis. After pyrolysis, the sample is naturally cooled to room temperature. Finally, the biochar is collected directly in the quartz tube; Condensation products are collected into glass bottles by ice water (1°C) condenser; After drying, the non-condensable gas is collected in a gas collection bag. In order to ensure the reliability of experimental data and the accuracy of pyrolysis product analysis, each group of experiments were repeated for more than 3 times.

### 2.5 Sample characterization and analysis methods

#### 2.5.1 Sample characterization

The contents of C, H and N elements in TS samples before and after pretreatment were determined by elemental analyzer, and the contents of O elements were calculated by subtraction method.

The industrial analysis of ash content, volatile matter, and fixed carbon in the samples was conducted according to the national standard GB/T 28731—2012 (GB/T: Chinese abbreviation for China Recommended National Standard).

The concentrations of inorganic elements (alkali metal K, alkaline earth metals Mg and Ga, and inorganic element Cl) in the TS samples before and after pretreatment were determined using ICP-OES. The inorganic element removal rate of TS samples after pretreatment was calculated according to Equation 1:

$$\alpha_{p}=\frac{m_{c}-m_{e}}{m_{c}}\times 100\%$$ (1)

Where $\alpha_{p}$ is the inorganic element removal rate of TS samples after pretreatment, %; *m*_*c*_ and *m*_*e*_ denote the concentration of inorganic elements in the original samples and the pretreated samples, respectively, mg/kg.

#### 2.5.2 Yield analysis of pyrolysis products

The yield of three-phase products (bio-oil, biochar, gas) obtained by pyrolysis of TS before and after pretreatment was calculated according to Equations 2–4:

$$Y_{bio-oil}=\frac{m_{bio-oil}}{M}$$ (2)

$$Y_{biochar}=\frac{m_{biochar}}{M}$$ (3)

$$Y_{gas}=\frac{M-{m_{biochar}-m}_{bio-oil}}{M}$$ (4)

Where *Y*_*bio-oil*_, *Y*_*biochar*_ and *Y*_*gas*_ are respectively the yield of bio-oil, biochar and gas obtained by pyrolysis of TS before and after pretreatment, %; *M* is the mass of the samples before pyrolysis, g; *m*_*bio-oil*_ and *m*_*biochar*_ are respectively the mass of bio-oil and biochar, g.

#### 2.5.3 Analysis of bio-oil compounds

The effects of different pretreatment methods on chemical components of bio-oil were analyzed by GC-MS. The identification of compounds in bio-oils was based on mass spectrometry data and confirmed by comparison with the NIST 2011 mass spectrometry database. The relative content of each compound was expressed as GC peak area percentage by peak area normalization method.

GC conditions: Chromatographic column: HP-5MS capillary column (30.0 m × 0.25 mm × 0.25 μm). Carrier gas: Helium (≥99.999%). Carrier gas flow rate: 1.0 mL/min. Injection mode: Split injection; Split ratio: 50:1. Injector temperature: 290°C. Heating procedure: 40°C for 5 min, 5 °C/min rate of heating up to 280°C, maintain 15 min.

MS conditions: Ionization scanning mode: Transmission line temperature: 230°C; Ionization energy: 70 eV; EI ion source temperature: 230°C; Quadrupole temperature: 150°C; Quality scanning range: 30–350 amu.

#### 2.5.4 Analysis of physical properties of bio-oil

As described in reference (Yang et al., 2015), the water content of the bio-oil was determined using the Karl Fischer titration method in accordance with ASTM E203. Ash content was measured by quantifying the non-combustible residue in the bio-oil, following ASTM D482. The higher heating value (HHV) of the bio-oil was determined using a calorimeter, while the pH was measured with a pH meter.

## 3 Results and discussion

### 3.1 Effect of pretreatment on characteristics of TS

#### 3.1.1 Ultimate and Proximate analysis

The fuel quality of biomass before and after pretreatment are listed in Table 2. The ultimate analysis results showed that compared with raw material (TS) and single acid washing pretreatment (WTS), the combined pretreatment significantly increased the C content and decreased the O content and O/C molar ratio. Specifically, at the same torrefaction temperature, torrefaction-acid washing (T-W) increases C content and reduces O content more effectively than acid washing -torrefaction (W-T) pretreatment. In addition, the higher the torrefaction temperature, the more significant the removal effect of O element. For example, when the torrefaction temperature reaches 300°C, compared with TS, the content of C in HTS-W sample increases from 37.07% to 62.19%, the content of O decreases from 54.83% to 28.14%, and the molar ratio of O/C decreases from 1.48 to 0.08. These results indicate that torrefaction treatment has a significant effect on the removal of O element. Studies (Abelha and Kiel, 2020) have shown that during the torrefaction process at 200°C–300°C, lignin, cellulose and hemicellulose in tobacco stem biomass decompose, and O element is effectively removed through dehydration, decarboxylation and dehydroxylation reactions. In addition, torrefaction in this temperature range can also promote the catalytic action of alkaloids (nicotine, niacin, nicotinic acid, etc.) and the decomposition of carbohydrates, thus causing the occurrence of decarboxylation and decarbonylation reactions, and removing a large number of O elements (Yıldız and Ceylan, 2019; Mong et al., 2021). N element in tobacco stems mainly exists in the form of nitrogen-containing compounds such as protein, amino acid and nicotine (Yan et al., 2018). Compared with TS, the content of N element in all pre-treated samples has decreased, which helps to reduce the generation of nitrogen oxides in the subsequent pyrolysis process, thus reducing the risk of air pollution.

**TABLE 2:** The biomass properties of original and pretreated TS.

Sample	Ultimate analysis^a^ (wt%)				Proximate analysis^a^ (wt%)			O/C	H/C	HHV^b^ (MJ/kg)
	C	H	N	O^c^	Ash	Volatile	Fixed carbon			
TS	37.07	5.52	2.58	54.83	18.00	64.75	17.25	1.48	0.15	13.36
WTS	32.59	3.23	1.83	62.35	2.01	83.01	14.98	1.91	0.10	8.67
LTS-W	47.80	4.68	2.30	45.22	2.92	73.79	23.29	0.95	0.10	17.43
MTS-W	54.19	5.49	3.39	36.93	3.33	68.08	28.59	0.68	0.10	21.45
HTS-W	62.19	5.01	4.66	28.14	3.89	52.28	43.83	0.45	0.08	24.55
W-LTS	44.73	5.78	1.33	48.16	3.25	78.78	17.97	1.08	0.13	17.36
W-MTS	47.66	5.35	1.59	45.40	4.13	74.12	21.75	0.95	0.11	18.14
W-HTS	52.93	5.09	1.89	40.09	4.74	64.22	31.04	0.76	0.10	20.20

^a^ Dry basis.

^b^ Higher heating value on dry basis.

^c^ Calculated by difference.

Pretreatment also has a certain effect on the quality of biomass fuel. The ash content of all pre-treated samples decreased significantly due to the removal of a large number of inorganic substances from the tobacco stems by acid washing. The ash content of WTS decreased significantly from 18% to 2.01%. It is worth noting that at the same torrefaction temperature, the ash content of W-T pre-treated samples is higher than that of T-W samples, which is due to the rapid decomposition of organic components in the torrefaction process after acid washing, leading to the re-enrichment of ash (especially AAEMs) (Abelha and Kiel, 2020). The content of ash, fixed carbon and HHV increased with the increase of temperature, while the volatile content decreased. Of all the samples, HTS-W had the highest HHV at 24.55 MJ/kg. This trend is attributed to the fracture of the less heat-stable side chains (such as carbonyl, hydroxyl, carboxyl, etc.) in the hemicellulose and lignin in the tobacco stem during the torrefaction process, which leads to the reduction of volatiles. At the same time, with the deepening of torrefaction degree, the cross-linking and carbonization reactions further transformed the C element in lignin into fixed carbon, thus significantly increasing the fixed carbon content (Gao et al., 2022).

Overall, the combined pretreatment combines the advantages of torrefaction deoxidation and acid washing deashing, effectively overcoming the ash enrichment caused by torrefaction (especially AAEMs) and the increase of oxygen content caused by acid washing, and provides a feasible solution for optimizing the fuel characteristics of tobacco stem biomass.

#### 3.1.2 ICP-OES analysis

Previous studies have found that torrefaction may cause coking of AAEMs on the surface of the catalyst, thereby deactivating the catalyst and further inhibiting the formation of aromatic compounds (Cen et al., 2019; Chen et al., 2017a; Bhatnagar et al., 2022; Hu et al., 2023) impregnated tobacco leaves with AAEMs and found that the increase in K+ concentration increased the coke yield significantly, while promoted the conversion of dehydrated sugar to ketone in bio-oil. (Zeng et al., 2022) reported that the removal of AAEMs would promote the directional pyrolysis of hemicellulose into anhydrous sugar, additionally, Mg has a high catalytic activity for inhibiting the generation of aromatics. The addition of Ca usually affects the temperature of the maximum degradation rate during pyrolysis (Eom et al., 2012). Therefore, it is necessary to study the influence of different pretreatment methods on the content of AAEMs elements in TS.

**FIGURE 3:**
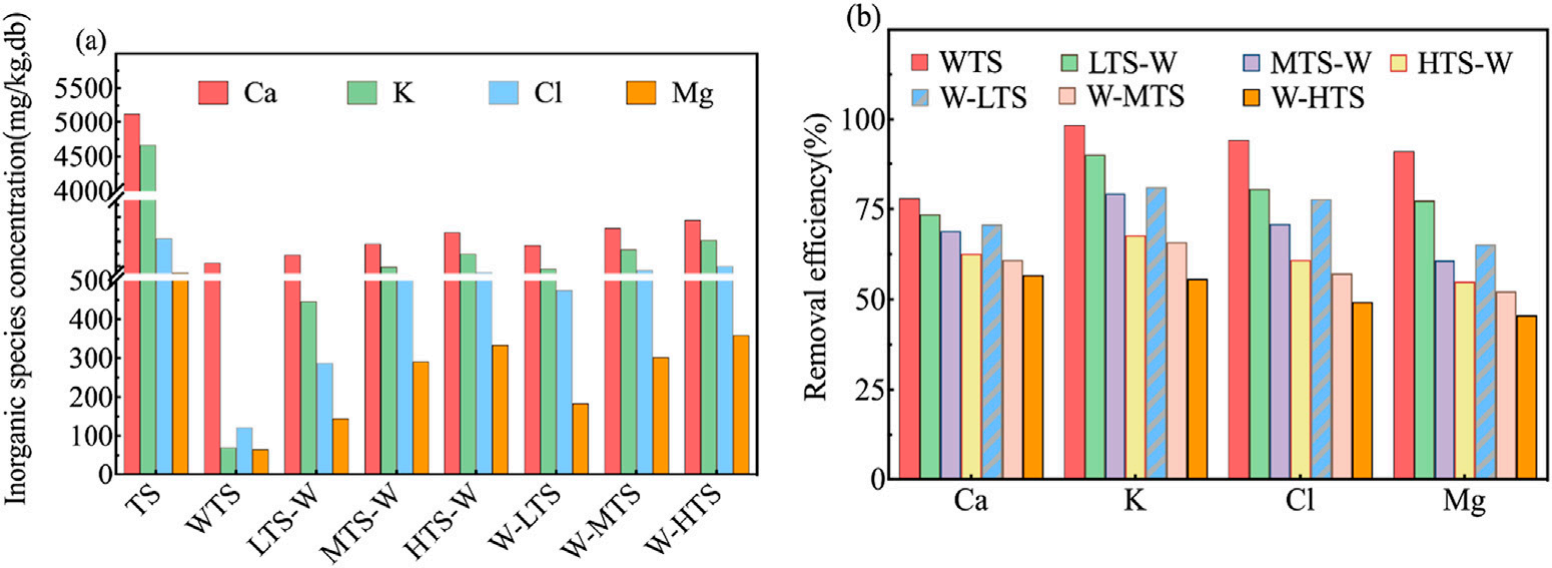
Inorganic species concentrations before and after pretreatment **(a)** and removal efficiency of inorganic species during pretreatment process **(b)** in TS samples.

Figure 3 illustrates the concentration of inorganic elements (a) and the removal rate of inorganic elements (b) in TS samples before and after pretreatment. The ICP-OES analysis revealed that the primary inorganic elements in TS are the alkali metal K, the alkaline earth metals Ca and Mg, and the inorganic element Cl. Ca and K were the highest inorganic elements in tobacco stem materials, and their concentrations were 5,130 mg/kg and 4,668 mg/kg, respectively.

Acid washing pretreatment had a remarkable effect on the removal of inorganic elements, and the removal rates of K, Cl and Mg were more than 90% (K: 98.5%, Cl: 94.3%, Mg: 91.2%). However, the removal effect of Ca is relatively poor, mainly because Ca usually exists in the form of calcium carbonate or silicate of calcium that is insoluble in organic acids (Ding et al., 2024), resulting in a limited ability of acetic acid washing to remove it.

The torrefaction temperature also has a certain effect on the removal effect of inorganic elements. With the increase of temperature, the removal rate of inorganic elements in the sample decreases, which is due to the occurrence of cross-linked carbonization during the torrefaction process, so that ash (especially AAEMs) is enriched, thus weakening the removal effect of acid washing. In addition, the removal effect of inorganic elements was different in different joint pretreatment sequences. Specifically, at the same torrefaction temperature, the inorganic element removal rate of T-W pretreatment (LTS-W, MTS-W, HTS-W) was significantly higher than that of W-T pretreatment (W-LTS, W-MTS, W-HTS), which was consistent with the change trend of ash content in industrial analysis results. This phenomenon may be attributed to the fact that, during T-W pretreatment, the hemicellulose in TS biomass decomposes following torrefaction pretreatment, while lignin undergoes partial softening and rearrangement. These changes render the biomass structure more porous and increase internal pore volume. Consequently, AAEMs originally encapsulated within the dense biomass structure become exposed, thereby enhancing their contact area with the subsequent acetic acid washing solution. For instance, voids generated by hemicellulose decomposition facilitate the penetration of the acid solution into the biomass interior, enabling it to react with metal elements and thus improving removal efficiency. Conversely, in W-T pretreatment, despite the initial acid washing process removing some water-soluble AAEMs, the subsequent high-temperature torrefaction process leads to secondary enrichment of residual AAEMs due to volatile matter migration, resulting in suboptimal removal performance.

Overall, the combined pretreatment effectively overcame the ash enrichment problem caused by torrefaction, not only optimized the physicochemical properties of tobacco stem biomass, but also helped to avoid the adverse effects caused by ash in the subsequent heat conversion process.

### 3.2 Effect of pretreatment on products yields

**FIGURE 4:**
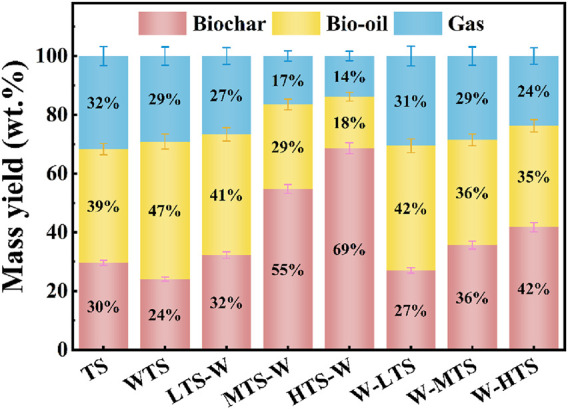
Mass yields of three phase products from pyrolysis of TS samples at 550°C before and after pretreatment.

The yields of three-phase products (biochar, bio-oil, gas) obtained by pyrolysis of tobacco stem samples before and after pretreatment at 550°C are shown in Figure 4. After acid washing, the yield of bio-oil increased significantly from 38.71% to 46.79%, while the yield of biochar decreased significantly from 29.63% to 24.08%. The results of ICP-OES analysis show that acid washing can effectively remove a large amount of AAEMs, reduce its catalytic action, inhibit the coke reaction and improve the yield of liquid products.

However, compared with acid washing pretreatment alone, the bio-oil yield of combined pretreatment was generally decreased (17.55%–42.44%), while the biochar yield was significantly increased (27.00%–68.62%). In addition, with the increase of torrefaction temperature, the yield of bio-oil and gas decreased, while the yield of biochar increased. This phenomenon can be attributed to several factors.

First, the torrefaction process leads to the early degradation of volatile components. Hemicellulose and cellulose, the primary precursors of bio-oil, are largely decomposed in the torrefaction process at 200°C–300°C, so the effective components that can be converted into bio-oil during subsequent high-temperature pyrolysis are reduced, resulting in a decline in bio-oil yield.

Second, the catalytic activity of inorganic elements contributes to ash enrichment during torrefaction, while AAEMs can catalyze the coking reaction in the carbonization process (Haddad et al., 2017). Specifically, the ring-opening properties of K element and the presence of Ca and Mg elements accelerate the carbonization of pyrolytic intermediates, thereby driving a significant increase in biochar yield.

Furthermore, torrefaction alters the physicochemical properties of biochar. The increased specific surface area and pore volume of biochar after torrefaction facilitate secondary reactions between bio-oil and biochar, thus reducing the yield of bio-oil (Chen et al., 2012).

The combined pretreatment sequence also has a significant effect on the yield of the product. At the same torrefaction temperature, the yield of bio-oil and gas of W-T combined pretreatment samples was significantly higher than that of T-W combined pretreatment samples. Specifically, the bio-oil yield from high to low was W-LTS (42.44%) > LTS-W (41.09%) > W-MTS (35.86%) > W-HTS (34.55%) > MTS-W (28.75%) > HTS-W (17.55%). The results showed that acid washing before torrefaction was more beneficial to increase the yield of bio-oil.

### 3.3 Effect of pretreatment on properties of bio-oil

#### 3.3.1 Effect of pretreatment on chemical composition of bio-oil

**FIGURE 5:**
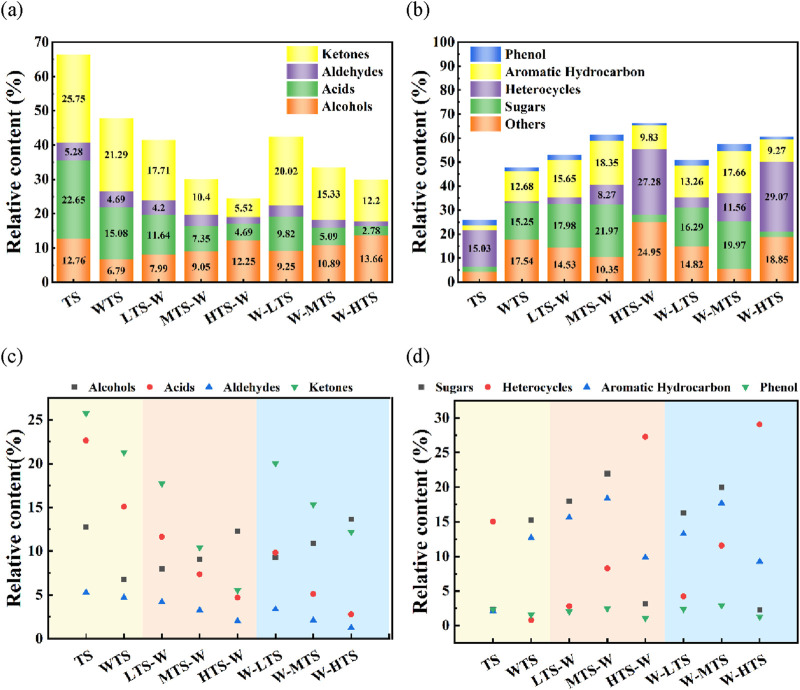
Distribution of bio-oil compounds under different pretreatment conditions: **(a,c)** oxygen-containing compounds (ketones, aldehydes, acids, alcohols); **(b,d)** phenols, aromatic hydrocarbons, heterocyclic and other compounds.

The chemical composition of bio-oil under different pretreatment methods was analyzed by GC-MS, and more than 200 compounds were detected. These compounds are mainly produced by the decomposition of hemicellulose, lignin, cellulose, alkaloids, pectin, starch and protein in tobacco stems at high temperatures (Ma et al., 2023a). To facilitate the analysis, these compounds were divided into 10 groups according to their structural characteristics: phenols, acids, ketones, aldehydes, alcohols, aromatic hydrocarbons, heterocycles, sugars and others. Figure 5 shows the composition distribution of compounds in the tobacco stem bio-oil sample, where (a, c) represents the content of oxygen-containing compounds (ketones, aldehydes, acids, alcohols), and (b, d) represents the content of phenols, aromatic hydrocarbons, heterocycles, and other compounds.

The results showed that pretreatment had a significant effect on the distribution of compounds in bio-oil, and the change of compound components in bio-oil was closely related to the removal of AAEMs. In addition, in the process of acetic acid washing, the depolymerization of cellulose and hemicellulose will also cause changes in the type and content of compounds (Li et al., 2022). The cross-linked carbonization that occurs during torrefaction causes AAEMs to accumulate again, and the decomposition of macromolecules such as hemicellulose, cellulose, and lignin in the range of 200°C–300°C during torrefaction also affects the distribution of compounds. The details are as follows.

##### 3.3.1.1 Oxygen compounds (ketones, aldehydes, acids, alcohols)

Carbonyl compounds (ketones, aldehydes, carboxylic acids and their derivatives) can significantly affect the quality and stability of bio-oils and limit their application (Oh et al., 2021). Therefore, reducing the content of these compounds is very important to improve the quality of bio-oil. As shown in Figures 5a,c, the contents of ketones, aldehydes and acids in bio-oil decreased significantly no matter what pretreatment method was adopted.

The ketone content of all compounds of TS bio-oil is the highest, which is 25.75%. After acid washing, its content is reduced to 21.29%, which may be due to the esterification and acidolysis of acetic acid with ketones to produce alcohols, acetic esters and other compounds. In the combined pretreatment samples, with the increase of torrefaction temperature, the decarbonylation reaction was enhanced, and the ketones were further reduced. When the temperature reached 300°C, the ketone content of the HTS-W sample dropped to 11.52%. The reduction of ketones means that a large amount of oxygen is removed, indicating that the stability of the bio-oil is improved. In addition, decarbonylation can also promote the reduction of aldehydes (Zhang et al., 2017).

The acids obtained from the pyrolysis of tobacco stem mainly come from the acetic acid produced by deacetylation of hemicellulose (Sun et al., 2019). After acid washing pretreatment, the acid content decreased from 22.65% of TS to 15.08% of WTS. In the combined pretreatment, the acid content of W-HTS sample decreased further with the increase of torrefaction temperature, and the acid content of W-HTS sample was the lowest, only 2.78%. This is due to the decomposition of hemicellulose and part of cellulose during torrefaction and acid washing, resulting in a significant reduction in the content of acid as hemicellulose or cellulose derived compounds during subsequent pyrolysis. The presence of acid will corrode equipment, reduce catalyst activity, and accelerate the aging of bio-oil, so its removal is of great significance for the upgrading of bio-oil (Chen et al., 2017b).

The representative product of alcohol compounds in the bio-oil of tobacco stem pyrolysis is methanol, which can be used as a clean energy instead of traditional fuel. The results showed that, compared with raw materials, the alcohol content decreased after acid washing pretreatment, while the alcohol content increased gradually with the increase of torrefaction temperature in combined pretreatment.

##### 3.3.1.2 Phenolic compounds

Phenols are the main products of lignin depolymerization (Ma et al., 2023b), which have important application value in chemical and pharmaceutical fields. For example, catechols have anti-inflammatory and antioxidant properties, and 2-methoxyphenol is widely used in the synthesis of anti-cold drugs. After combined pretreatment, the content of phenol increased from 9.93% of TS to 11.95% of W-MTS. It is worth noting that the phenol content increases first and then decreases with the increase of torrefaction temperature. This is because under the torrefaction conditions of 220°C–260°C, the condensation and cyclization reaction of ketones and other carbohydrates can generate more phenols, and when the temperature continues to rise, the high temperature promotes the secondary cracking of phenols, which is converted into CO, CO_2_, H_2_ and other gases, so that the phenol content is reduced.

##### 3.3.1.3 Aromatic hydrocarbon compounds

The representative products of aromatic hydrocarbons in the pyrolysis oil of tobacco stem include xylene, ethylbenzene, alkyl benzene and polycyclic aromatic hydrocarbons, which are mainly derived from the dehydration, decarbonylation and aromatization reactions of oxygen-containing compounds (Mong et al., 2021; Sun et al., 2019). After acid washing pretreatment, aromatics content increased significantly from 2.09% of TS to 12.68% of WTS. In this experiment, the best condition of aromatics formation is MTS-W, and the yield is as high as 18.35%. This is mainly due to the reduction of secondary cracking reactions during pyrolysis after the removal of AAEMs by acid washing, thus promoting the generation of aromatic hydrocarbons. In addition, in the lower temperature torrefaction process, the cyclic and deoxidation reactions also promote the conversion of ketones and aldehydes into a large number of aromatic compounds (Wang et al., 2015). However, when the torrefaction temperature is increased to 300°C, the aromatics content decreases significantly, which is due to excessive cracking or carbonization caused by high temperature, which enhances the heat-derived Cox and coke, which in turn inhibits the generation of aromatic hydrocarbons.

##### 3.3.1.4 Heterocyclic compounds

The heterocyclic compounds were almost completely removed after acid washing, indicating that most of the heterocyclic compounds were effectively dissolved during the washing process. In combined pretreatment, the content of heterocyclic compounds increased significantly when the torrefaction temperature increased from 260°C to 300°C. Among all pretreatment methods, the best condition of heterocyclic compound formation was W-HTS, and the yield was as high as 29.07%. This is due to the fact that nicotine, as a special nitrogen-containing heterocyclic compound in tobacco stem, is pyrolyzed at high temperatures to produce nitrogen-containing pentane and six-membered heterocyclic products, which continue to react with hydrocarbon free radicals to further produce pyridine, pyrrole, pyrazine and piperidine nitrogen-containing heterocyclic compounds (Zhang et al., 2017).

##### 3.3.1.5 Sugars compounds

The main representative product of sugar in bio-oil of tobacco stem is L-glucan (LGA). After acid washing, the sugar content increases significantly from 2.35% to 15.25%. This is because the polymerization degree of cellulose in tobacco stem is reduced after acetic treatment, and C6-hydroxyl group and B-1, 4-glucoside bond are broken to produce C1 cation, thus producing a large amount of LGA (Zhang et al., 2011). Among all pretreatment methods, the content of carbohydrate in MTS-W pyrolysis bio-oil was the highest (21.97%). As the torrefaction temperature continues to rise to 300°C, the sugar content drops sharply, due to the high temperature conversion of sugar, as a precursor of nitrogenous compounds, into high value nitrogenous compounds such as imidazole, pyridine, and pyrrole, which also coincides with the significant increase in heterocyclic compound content at 300°C. At present, LGA has a high application value in the field of biomedicine and fine chemical industry, which has medicinal value in the treatment of inflammatory intestinal diseases, hepatic encephalopathy, reducing blood cholesterol, etc. In addition, LGA can also be used in microbial fermentation and as a carbon source to produce ethanol and succinic acid.

#### 3.3.2 Effect of pretreatment on distribution of high-value compounds in bio-oil

**FIGURE 6:**
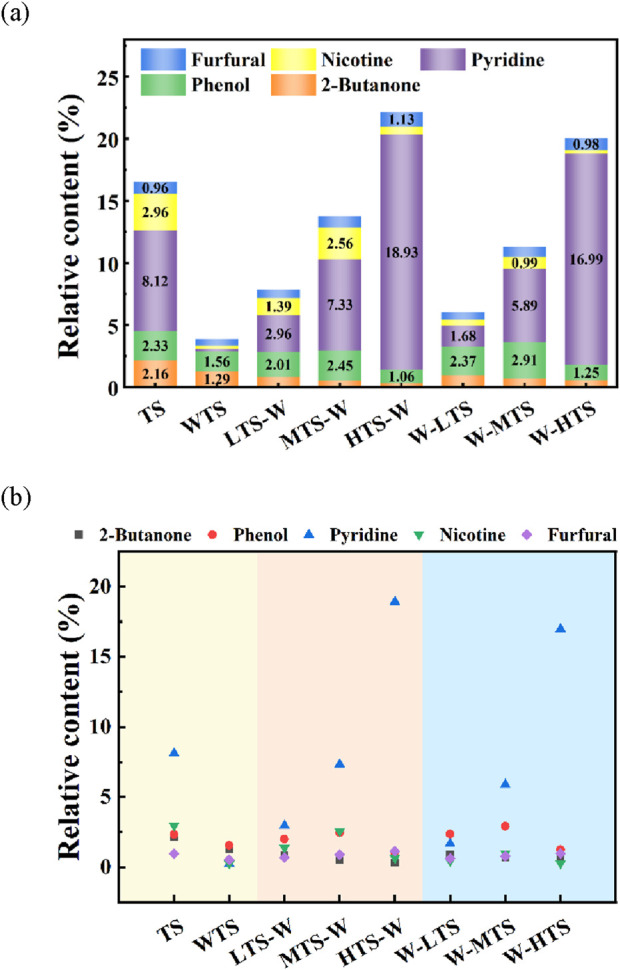
Distribution of high-value compounds in TS bio-oil: **(a)** specific content; **(b)** contrast effect.

As the auxiliary raw material of cigarette formulation, it is of great significance to study the representative compounds in the bio-oil of tobacco stem pyrolysis for improving its compatibility and application value with cigarette raw material. Tobacco stem bio-oil contains furfural, phenol, 2-butanone, pyridine, nicotine and other compounds, which have high alkalinity and unique aroma characteristics. Noteworthily, in this study, high-value compounds were defined as i) Chemical precursors in bio-oils that can be used in cigarette processing and production, such as compounds that can increase nicotine and flavor components in tobacco products; ii) Feedstocks that can be widely applied in medical, agricultural and chemical production. Due to the high market demand for these compounds and their potential to replace fossil-based raw materials in the chemical industry, they also have significant economic value. Figure 6 shows the component distribution of high-value compounds in the pyrolysis bio-oil of tobacco stem samples.

Furfural (C_5_H_4_O_2_) has been identified as one of the 30 most valuable compounds (Cardoso and Ataide, 2013) because of its unique sweet odor, which is often used in the tobacco industry to improve the odor of cigarettes when lit. Furfural is formed by dehydration, decarboxylation and cyclization of oligosaccharides from the pyrolysis of cellulose, hemicellulose and pectin macromolecules in tobacco stem. The results showed that after combined pretreatment, the furfural content reached the highest value of 1.13% in HTS-W, which was due to the further cyclization and dehydration of 3-deoxyglucose, 1, 4-dehydro-dihydroxypyranose and D-xylose produced by the cracking of cellulose and pectin to form furfural at 300°C (Ge et al., 2015).

Nicotine (C_10_H_14_N_2_), as a special nitrogen-containing compound in tobacco stem, can be widely used in agriculture and medical fields after extraction. After acid washing, nicotine content decreased significantly from 2.96% to 0.26%. In combined pretreatment, nicotine content increased first and then decreased with the increase of torrefaction temperature. This trend indicates that low temperature is conducive to the precipitation of nicotine, and further warming will promote the decomposition of nicotine into pyridine, pyrrole, pyrazine and other hot pentyclic nitrogenous compounds, which is also consistent with the significant increase in pyridine content at 300°C.

Pyridine (C_5_H_5_N) is a volatile organic compound in tobacco smoke, and its content and release characteristics have important effects on the composition and properties of tobacco smoke. Previous studies (Li and Hecht, 2022) have found that pyridine in tobacco smoke can cause respiratory diseases such as cough and asthma, and may be transformed into carcinogens through metabolism, thus causing significant harm to the health of smokers. It can be seen from this experiment that pyridine in WTS bio-oil is almost completely removed after acid washing. In combined pretreatment, the content of pyridine increased with the increase of torrefaction temperature. This increasing trend is mainly attributed to the fact that high-temperature torrefaction promotes the breakdown of nicotine, which further generates pyridine nitrogen-containing compounds.

As a multi-purpose hydroxybenzene compound, phenol (C_6_H_6_O) is widely used in medicine, agriculture, preparation of spices, dyes, resins and other industrial fields because of its anti-corrosion, sterilization, disinfection and other functions. After combined pretreatment with W-MTS, the phenol content increased from TS 2.33%–2.96%. This is because the pyrolysis products of lignin in tobacco stem contain a large number of phenolic compounds, such as cresol, 2-methoxyphenol, 2-methylphenol, etc. These phenolic compounds will undergo dehydration condensation, isomerization and alkylation reactions during torrefaction. Finally, phenol and its derivatives are formed. When the torrefaction temperature increased from 260°C to 300°C, the phenol content decreased significantly, which may be because the high temperature promoted the dehydroxylation reaction of phenol, formed water and oxygen-containing gas, and thus reduced the total amount of phenol.

2-butanone (C_4_H_8_O), the main component of ketone compounds in tobacco stem bio-oil, is commonly used as a flavor enhancer in cigarettes and e-cigarettes, which can not only regulate the taste of tobacco, rich taste, but also improve the burning performance of tobacco products. Consistent with the results of the ketone studies above, the content of 2-butanone decreased significantly after combined pretreatment due to the removal of O element (mainly decarbonylation) by torrefaction and ash removal by acid washing.

#### 3.3.3 Effect of combined pretreatment on chemical properties of bio-oil

In general, combined pretreatment can effectively regulate the chemical composition of bio-oil, reduce the unstable oxygen compounds, significantly increase the content of aromatic hydrocarbons and sugars, so as to improve the quality of bio-oil and expand its application potential. Specifically, acid washing treatment effectively removes pyridine and nicotine while reducing phenol and 2-butanone levels. In the combined pretreatment, the oxygen compounds (ketones and acids) in the pyrolysis bio-oil of HTS-W sample were the lowest, which indicated that the stability of bio-oil was improved. The combined pretreatment sequence showed obvious difference in the distribution of compounds. The T-W method was helpful to increase the yield of carbohydrate and aromatic compounds. The W-T method helps to increase the content of high-value nitrogen compounds and phenol, while reducing the content of harmful compounds such as acid and pyridine. In addition, high-temperature torrefaction can promote the thermal cracking of nicotine, and further generate pyridine compounds.

#### 3.3.4 Effect of pretreatment on physical properties of bio-oil

Low ash content, low moisture, weak acidity and high HHV are key factors to improve the stability and application potential of bio-oil. Table 3 shows the changes of HHV, water content, pH value and ash content of pyrolytic bio-oil of tobacco stem samples before and after pretreatment. The results showed that compared with raw material TS, all pretreatment processes effectively reduced the moisture and ash content of bio-oil, and significantly increased its pH and HHV. Among them, HTS-W bio-oil HHV significantly increased from TS 16.03%–24.06%, pH significantly increased from 2.96% to 3.37%. The water removal effect of W-HTS was the best, which decreased significantly from 48.56% to 29.25%. The bio-oil obtained from WTS has the lowest ash content, with a value of 0.04%. This is mainly due to the high efficiency of acetic acid washing pretreatment to remove AAEMs in tobacco stems, resulting in a significant decrease in ash content in pyrolysis products. However, it is worth noting that with the strengthening of the degree of torrefaction, the ash content showed a gradual upward trend.

**TABLE 3:** Physical properties of bio-oil samples.

Samples	HHV(MJ/kg)	Water content (wt%)	pH	Ash content (wt%)
TS	16.03	48.56	2.96	0.29
WTS	20.76	37.86	3.09	0.04
LTS-W	22.45	34.29	3.23	0.06
MTS-W	23.77	32.78	3.31	0.07
HTS-W	24.06	29.25	3.37	0.08
W-LTS	21.02	32.27	3.18	0.05
W-MTS	22.66	28.75	3.27	0.06
W-HTS	23.29	26.33	3.36	0.06

## 4 Conclusion

The effect of acid washing and torrefaction pretreatment on the quality improvement of tobacco stem biomass and bio-oil was analyzed. The main conclusions are as follows.

(1) Biomass properties were significantly modified by the pretreatment methods. Compared with the original TS material, the combined T-W pretreatment at 300°C significantly reduced the oxygen content in the biomass, from 54.83% to 28.14%, while HHV increased from 13.36 MJ/kg to 24.55 MJ/kg. W-T combined pretreatment at 220°C reduced the ash content of biomass from 18.00% to 2.92%. The removal rates of K, Cl, and Mg were 98.5%, 94.3% and 91.2%, respectively. These results show that combined pretreatment effectively avoids the re-enrichment of ash (especially AAEMs) during torrefaction, overcomes the problem of increased oxygen content caused by acid washing, and improves the fuel properties of biomass.
(2) Bio-oil yield was affected differently by various pretreatment methods. Compared with TS raw material, the yield of bio-oil pretreatment with acid washing increased from 38.71% to 46.79%. However, in the combined pretreatment, the bio-oil yield decreased with the increase in torrefaction temperature. At 220°C, the bio-oil yield was 42.44%, which was the highest value of all the combined pretreatment conditions. The results showed that the combined pretreatment with the addition of acid washing can effectively address the negative impact of the cross-linking carbonization reaction during the torrefaction process on the bio-oil yield.
(3) The distribution of bio - oil compounds underwent remarkable changes after combined pretreatment. After combined pretreatment, the content of high-value compounds increased significantly. Aromatics content increased from 2.09% to 18.35% of MTS-W. The content of heterocyclic compounds increased from 5.03% to 29.07% of W-HTS. The content of carbohydrate increased from 2.35% to 21.97% of MTS-W. At the same time, the content of acids, ketones and aldehydes, which negatively affect the stability of the bio-oil, decreased significantly. In HTS-W process, furfural content increased from 0.96% to 1.13%. In the W-MTS process, phenol content increased from 2.33% to 2.91%. In the W-HTS process, the pyridine content increased from 8.33% to 18.93%.
(4) The physical properties of bio-oil were notably improved by combined pretreatment. Compared with TS feedstock, combined pretreatment effectively improved the physical properties of bio-oil. All combined pretreatment processes significantly reduced the moisture and ash content of the bio-oil, while increasing its pH and HHV.

## Data Availability

The original contributions presented in the study are included in the article/supplementary material, further inquiries can be directed to the corresponding authors.
